# Genetic background influences arterial vasomotor function in male and female mice

**DOI:** 10.14814/phy2.15824

**Published:** 2023-09-28

**Authors:** Dylan Holly, Hyoseon Kim, Christopher R. Woodman, Michael P. Massett

**Affiliations:** ^1^ Department of Kinesiology and Sport Management Texas A&M University College Station Texas USA; ^2^ Department of Kinesiology and Sport Management Texas Tech University Lubbock Texas USA

**Keywords:** aorta, carotid artery, endothelium, femoral artery, inbred mice, sex differences

## Abstract

The purpose of this study was to assess the influence of genetic background and sex on nitric oxide (NO)‐mediated vasomotor function in arteries from different vascular territories. Vasomotor function was assessed in thoracic aorta, abdominal aorta, carotid arteries, and femoral arteries from the following mouse strains: SJL/J, DBA/2J, NZW/LacJ, and C57BL/6J. Contractile responses were assessed using the α1‐adrenergic receptor agonist phenylephrine (PE, 10^−9^–10^−5^ M). Vasorelaxation responses were assessed by examining relaxation to an endothelium‐dependent vasodilator acetylcholine (ACh, 10^−9^–10^−5^ M) and an endothelium‐independent vasodilator sodium nitroprusside (SNP, 10^−9^–10^−5^ M). To evaluate the role of NO, relaxation responses to ACh and SNP were assessed in the absence or presence of a nitric oxide synthase inhibitor (*N* omega‐nitro‐l‐arginine methyl ester hydrochloride: 10^−4^ M). Vasomotor responses to ACh and PE varied across strains and among the arteries tested with some strains exhibiting artery‐specific impairment. Results indicated some concentration–response heterogeneity in response to ACh and SNP between vessels from females and males, but no significant differences in responses to PE. Collectively, these findings indicate that vasomotor responses vary by genetic background, sex, and artery type.

## 1 INTRODUCTION

Recent reports by the American Heart Association (AHA) indicate that 49% (~126.9 million) of the US population ≥20 years has some form of cardiovascular disease (CVD) (Tsao et al., 2022); approximately 44% (~60.8 million) are female (Tsao et al., 2022). However, important differences exist between males and females and their relative risk of CVD. Males typically develop CVD earlier in life and are at greater risk for coronary heart disease (Tsao et al., 2022). In contrast, women are at higher risk for stroke than men accounting for roughly 60% of annual stroke deaths (Bots et al., 2017), another disease rooted in vascular pathology. Thus, sex differences in the type and susceptibility to CVD exist. Among CVD risk factors, impaired endothelial function is a fundamental component of atherosclerosis and therefore, a precursor of overt CVD (Reddy et al., 1994; Zeiher et al., 1991).

The endothelium is critical for maintaining vascular tone via the release of vasoactive molecules that regulate calcium release, hemostatic balance, permeability, angiogenesis, and cell survival (Aird, 2007; Vanhoutte, 1989). Sex differences in endothelial function, measured as flow‐induced dilation, have been reported throughout the lifespan (Celermajer et al., 1994). Because endothelial dysfunction occurs at a later age in women, sex differences in endothelial function have been attributed to the protective effects of estrogen (Celermajer et al., 1994; Gilligan et al., 1994). Sex differences in endothelial function have been observed in blood vessels isolated from rodents, although these results are inconsistent. Greater endothelium‐dependent responses to acetylcholine (ACh) were observed in mesenteric arteries from adult (27‐week‐old) and older (57‐week‐old) female mice (Ogola et al., 2022). Conversely, no sex differences were reported for responses to ACh in mesenteric arteries from young (4–9 months) or old (23–32 months) C57BL6/J (B6) mice (Cole et al., 2022). In thoracic aorta (TA) from male and female ICR strain mice, endothelial responses to ACh were similar at 5 months of age, but greater in females at 21 months of age (Takenouchi et al., 2009). The variable reports of sex differences in endothelial function in blood vessels from mice might be related to vessel type or mouse strain.

Although endothelial dysfunction is associated with a number of CVD risk factors, there is strong evidence that genetic factors play a major role in susceptibility to CVD (Nikpay et al., 2015) and other related vascular pathologies such as hypertension and atherosclerosis (Kauko et al., 2021; Paigen et al., 1985, 1990; Samani et al., 2007; Warren et al., 2017). Previous studies in inbred mice support the contribution of genetic background to differences in susceptibility to CVD. Paigen et al. reported mouse strain differences in atherosclerosis in the ascending aorta (1985, 1990). Vascular remodeling in response to changes in blood flow in the common carotid artery (CA) also varies across multiple strains of inbred mice (Harmon et al., 2000; Korshunov & Berk, 2004). Our laboratory previously reported a strong correlation between genetic background and vasomotor function in TA across 27 inbred strains of male mice (Kim et al., 2016). Significant strain‐dependent differences in both maximal responses and sensitivity to the α_1_ adrenergic receptor agonist phenylephrine (PE) and membrane depolarizing agent potassium chloride (KCl) were reported. Maximal responses to the endothelium‐dependent vasodilator ACh also varied by mouse strain. Strain differences in vasomotor response varied two to fivefold. Blunted vasorelaxation responses were likely due to impaired endothelial function since relaxation responses to sodium nitroprusside (SNP), an endothelium‐independent agonist, were not different (Kim et al., 2016). Collectively, these findings suggest that genetic background influences vasomotor function in mouse aorta.

Although vascular function is commonly measured in the TA, that artery might not be representative of the entire vascular tree (Kleinbongard et al., 2013; Leloup et al., 2015; Potente & Makinen, 2017; Ryan et al., 2002). In addition, little is known about the genetic regulation of vascular function beyond the TA. Therefore, the aims of this study were to (1) assess the influence of genetic background on vasomotor function in arteries from different locations in the arterial tree; (2) identify sex differences in vasomotor function; and (3) determine the importance of nitric oxide (NO) in vasorelaxation response in male and female mice with different genetic backgrounds. We hypothesized that NO‐mediated vasorelaxation in the abdominal aorta (AA), CA, and femoral artery (FA) would be impaired in strains with previously documented impairment in the TA. Furthermore, we hypothesized that NO‐mediated vasorelaxation would be impaired in arteries from male mice when compared to arteries from female mice.

## 2 MATERIALS AND METHODS

### 2.1 Ethics approval

Prior to initiating this study, approval was received from the Texas A&M University Institutional Animal Care and Use Committee. All procedures were performed under the Public Health Service's Policy on Humane Care and Use of Laboratory Animals guidelines.

### 2.2 Animals

Male and female mice from four inbred strains (C57BL/6J #000664, DBA/2J #000671, NZW/LacJ #001058, SJL/J #000686) were purchased from Jackson Laboratories and housed at the Texas A&M Comparative Medicine Program Facility. All mice were received at 5–8 weeks of age. Mouse strains were chosen based on phylogenetically distinct background and previous reports of impaired endothelium‐dependent relaxation in the TA (Chen et al., 2007; Kim et al., 2016). Vascular studies were conducted when mice were 10–12 weeks of age to match our previous studies (Chen et al., 2007; Kim et al., 2016). Mice were housed in the same room under standard conditions (non‐barrier), maintained on a 12:12 h light–dark cycle in a controlled temperature (21.0–22.0°C), and allowed food (Teklad Rodent Diet, 8604) and water ad libitum. Mice were examined daily for health concerns by Texas A&M Animal Care Facility veterinarians or staff.

### 2.3 Vasoreactivity assessment

#### 2.3.1 Isolation of arteries

At 10–12 weeks of age, mice were weighed and anesthetized by intraperitoneal injection of ketamine (80 mg/kg) and xylazine (5 mg/kg). TA, AA, CA, and FA were isolated. Connective tissue and perivascular adipose tissue were carefully removed in ice‐cold physiological saline solution pH 7.4 (in mM: 118.31 NaCl, 4.69 KCl, 1.2 MgSO_4_, 1.18 KH_2_PO_4_, 24.04 NaHCO_3_, 0.02 EDTA, 2.5 CaCl_2_, and 5.5 glucose) under a microscope. Arteries were cut into 2 mm ring segments of equal length, and each ring segment was suspended in an organ chamber (DMT610M or 620M Multi Chamber Myograph System; Danish Myo Technology) filled with 8 mL of oxygenated (95% O_2_, 5% CO_2_) physiological saline solution and allowed to equilibrate at 37°C for at least 30 min.

#### 2.3.2 Functional evaluation

Optimal resting tension was determined following standard normalization procedures for wire myography (del Campo & Ferrer, 2015; Leloup et al., 2015). Briefly, arterial rings were stretched in a stepwise fashion until the calculated transmural pressure reached 13.3 kPa (100 mmHg). Resting tension was set to an internal circumference of approximately 90% of that at 13.3 kPa. Two arterial rings from each type of artery were utilized (TA, AA, CA, FA); thus, eight artery segments were studied per animal. Arterial ring segments were treated with a single concentration of a non‐selective adrenergic receptor agonist norepinephrine (NE: 3 × 10^−7^ M) to confirm that the arteries were viable. Rings that contracted less than 20% relative to baseline tension were excluded from further experiments. Cumulative concentration–response curves to PE (a selective α1‐adrenergic receptor agonist, 10^−9^–10^−5^ M in full log increments) were generated to assess contractile function, whereas cumulative concentration–response curves to ACh, a muscarinic receptor agonist and SNP, an NO donor (10^−9^–10^−5^ M in half‐log increments) were generated to assess endothelium‐dependent and independent vasorelaxation, respectively. Prior to assessing relaxation responses to ACh and SNP, one ring segment from each group (TA, AA, CA, FA) was treated with an NO synthase (NOS) inhibitor *N* omega‐nitro‐l‐arginine methyl ester hydrochloride (l‐NAME: 10^−4^ M) for 30 min, whereas the non‐treated ring served as control. Concentration–response curves to ACh and SNP were generated after each ring was pre‐constricted with a concentration of PE eliciting 70% of maximal contraction for each vessel. Percent contraction responses were calculated as [(*D*_P_ – *D*_B_)/*D*_B_] × 100, where *D*_P_ is the maximal force generated by PE and *D*_B_ is the baseline force. Percent relaxation responses were calculated as [(*D*_P_ – *D*_D_)/(*D*_P_ – *D*_B_)] × 100, where *D*_P_ is the maximal force pre‐generated by PE, *D*_D_ is the lowest force generated at a given dose of ACh or SNP, and *D*_B_ is the baseline force. The half‐maximal effective concentrations (EC_50_) for ACh and SNP were calculated from cumulative concentration–response curves to each agent using the logarithmic dose–response with four‐parameter variable curve fitting (GraphPad Prism 8). Some of the concentration–response curves to PE failed to reach a plateau; therefore, EC_50_ for PE was not calculated. As an alternative area under the curve (AUC) for PE was determined for each vessel as a marker of potency and efficacy using GraphPad Prism 8.

### 2.4 Statistical analysis

All data are reported as means ± SEM. Eta squared (*η* ^2^) was used as a measure of effect size (Cohen, 1988). Thresholds for *η* ^2^ were set as small, *η* ^2^ = 0.01; medium, *η* ^2^ = 0.06; and large, *η* ^2^ ≥ 0.14 (Cohen, 1988). A full factorial repeated measures mixed model analysis followed by Tukey's post hoc test was used to evaluate the effects of strain and sex on concentration–response data for ACh, SNP, and PE (Muhammad, 2023). A two‐way analysis of variance (ANOVA) and Tukey's post hoc test, if appropriate, were performed to determine the effects of strain and sex on maximal vasoreactivity, AUC, and EC_50_ values. *t*‐Tests with Welch's correction were used to determine differences between l‐NAME and non‐l‐NAME treated vessels within each strain. If two rings from the same animal were used to determine vasoreactivity without l‐NAME present, the responses from the vessel segments were averaged before statistical analysis (Delp et al., 1995). If one ring was treated with l‐NAME and the other not, these rings were treated as individual rings. Outliers were detected using the ROUT method (*Q* = 1%) and removed if identified. Statistical significance was set at *p* ≤ 0.05 probability level. All statistical analyses were performed using GraphPad Prism 8.4.1 or JMP Pro 16 with Full Factorial Repeated Measures ANOVA Add‐In.

## 3 RESULTS

### 3.1 Endothelium‐dependent relaxation to ACh

**FIGURE 1:**
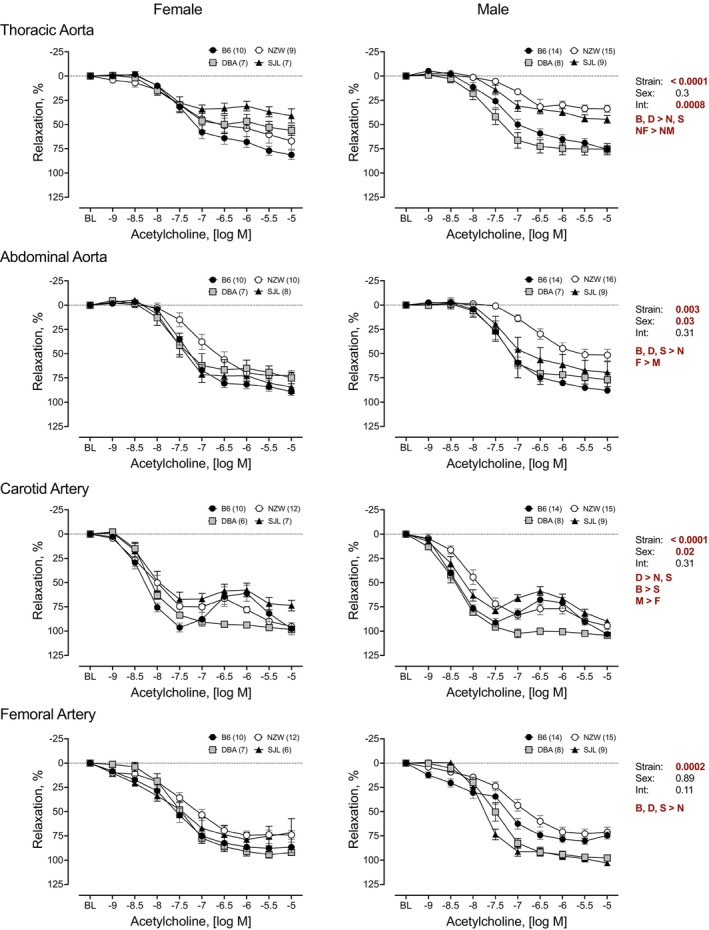
Strain and sex differences in endothelium‐dependent relaxation to acetylcholine (ACh) in thoracic and abdominal aorta, and carotid and femoral arteries. Cumulative concentration–response curves to ACh (10^−9^ to 10^−5^ M) were assessed in isolated arteries from female (*left*) and male (*right*) mice from four inbred strains, C57BL/6J (B6), DBA2/J (DBA), NZW/LacJ (NZW), and SJL/J (SJL). Cumulative concentration–response curves are expressed as percent relaxation (%). Main effects for strain, sex, and their interaction from a factorial repeated measures analysis are shown for each artery. The *p*‐value for concentration was significant for each artery (*p* < 0.0001 for all). Strain differences, determined by Tukey post hoc analysis, are indicated by letters B, B6; D, DBA; N, NZW; S, SJL. Sex differences are indicated by M, male; F, female. Males and females are plotted separately for clarity. Numbers in parentheses indicate the number of animals per strain for each artery. Values are expressed as mean ± SEM.

**FIGURE 2:**
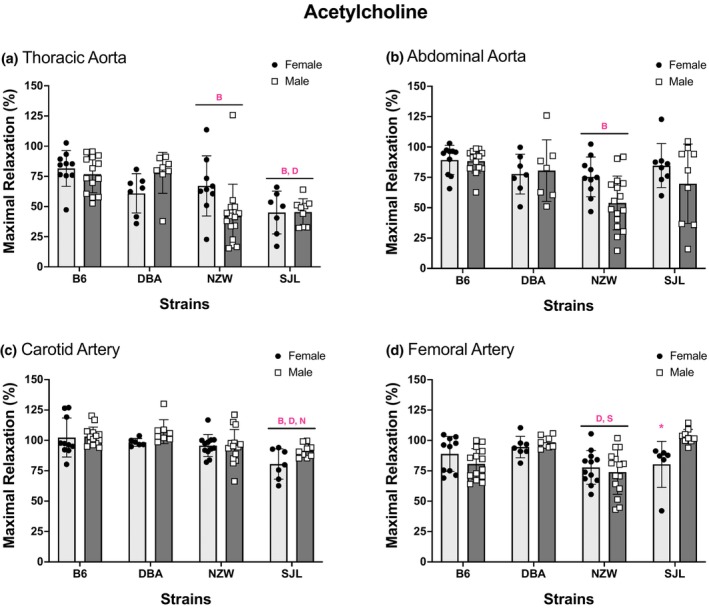
Maximal responses to acetylcholine differ by strain and sex. Values are expressed as mean ± SEM. Two‐way analysis of variance was used to determine the main effects of strain, sex, and interaction for (a) thoracic aorta (*p*_strain_ ≤ 0.0001, *p*_sex_ = 0.5, *p*_int_ = 0.01), (b) abdominal aorta (*p*_strain_ = 0.001, *p*_sex_ = 0.06, *p*_int_ = 0.2), (c) carotid artery (*p*_strain_ ≤ 0.0001, *p*_sex_ = 0.053, *p*_int_ = 0.3), and (d) femoral artery (*p*_strain_ ≤ 0.0001, *p*_sex_ = 0.25, *p*_int_ = 0.007). Strain differences, determined by Tukey post hoc analysis, are indicated by letters B, B6; D, DBA; N, NZW; S, SJL. *p < 0.05 significant pairwise comparison from strain by sex interaction. The number of animals per strain, sex, and artery is listed in Figure 1.

To determine the genetic contribution to endothelium‐dependent relaxation, cumulative concentration–response curves to ACh were performed in isolated TA, AA, CA, and FA from four strains of mice (Figure 1). All arteries from each strain relaxed in response to ACh. Repeated measures analysis identified significant strain differences for each artery. Responses in NZW were significantly lower than other strains for all arteries. Responses in arteries from SJL mice were significantly lower than other strains in the TA and CA. A significant main effect of sex was identified in the AA and CA. Responses were greater in AA from female mice, but smaller than males in CA. In the TA, a significant strain by sex interaction was observed and paired comparisons indicated a greater response in female NZW compared to male NZW. Maximal responses to ACh are shown in Figure 2. Two‐way ANOVA revealed significant strain differences in maximal responses to ACh (Max, %) across all arteries tested. Effect sizes (*η* ^2^) for strain were 0.29 for TA, 0.17 for AA, 0.26 for CA, and 0.29 for FA, indicating a large effect of strain for all arteries. Tukey's multiple comparison post hoc indicated that responses to ACh were significantly smaller in TA, AA, and FA from NZW compared with arteries from other strains (Figure 2). For example, maximal responses to ACh were approximately 25% lower in NZW aorta compared with aorta from B6. Relaxation responses to ACh in the TA and CA were lowest in SJL (Figure 2). In the TA, maximal responses in SJL were significantly lower than those in TA from B6 and DBA, with maximal responses <50% in aorta from both male and female mice. In CA, maximal responses to ACh were 10%–20% lower in SJL compared to all other strains. There were no significant sex differences for maximal responses to ACh. However, responses in females tended to be greater in AA (*p* = 0.06) and lower in CA (*p* = 0.053), similar to results observed in the concentration–response curves for those arteries. A significant strain by sex interaction was identified in FA with smaller responses in arteries from female versus male SJL. Overall, the effect of sex was small (*η* ^2^ < 0.05) for all arteries. Sensitivity as determined by EC_50_ (−log_10_ [*M*]), the concentration required to produce half‐maximal relaxation, is shown in Table 1. Significant strain differences in sensitivity (ACh EC_50_, −log_10_ [*M*]) were found in AA, CA, and FA (Table 1). Sensitivity was lowest in the NZW strain in all arteries. A significant sex difference in ACh EC_50_ was identified in AA with sensitivity being greater in arteries from female mice (Table 1).

**TABLE 1:** Half‐maximal relaxation concentrations (EC_50_) for acetylcholine (ACh).

Vessel/strain	Female	Male	*p*‐Value	Differences
Thoracic aorta				
B6 (10/14)	7.24 ± 0.12	7.33 ± 0.12	Strain: 0.06	
DBA (7/8)	7.23 ± 0.30	7.55 ± 0.13	Sex: 0.07	
NZW (8/14)	7.40 ± 0.17	6.79 ± 0.12	Int.: **0.005**	
SJL (6/9)	7.83 ± 0.09	7.20 ± 0.13		
Abdominal aorta				
B6 (10/14)	7.38 ± 0.07	7.36 ± 0.14	Strain: <**0.0001**	All > NZW
DBA (6/7)	7.60 ± 0.18	7.27 ± 0.14	Sex: **0.006**	F > M
NZW (10/16)	6.98 ± 0.15	6.66 ± 0.13	Int.: 0.37	
SJL (8/9)	7.55 ± 0.12	7.03 ± 0.16		
Carotid artery				
B6 (10/11)	8.42 ± 0.04	8.40 ± 0.06	Strain: **0.0007**	B6, DBA > NZW
DBA (7/8)	8.20 ± 0.06	8.44 ± 0.08	Sex: 0.32	
NZW (11/15)	8.10 ± 0.12	7.99 ± 0.11	Int.: 0.25	
SJL (6/7)	8.13 ± 0.16	8.31 ± 0.08		
Femoral artery				
B6 (10/13)	7.70 ± 0.14	7.65 ± 0.18	Strain: **0.0009**	B6, SJL > NZW
DBA (7/8)	7.57 ± 0.11	7.56 ± 0.10	Sex: 0.40	
NZW (10/14)	7.37 ± 0.12	7.09 ± 0.09	Int.: 0.86	
SJL (6/9)	7.85 ± 0.08	7.78 ± 0.06		

*Note*: Data are mean ± SEM; numbers in parentheses equal the number of females and males; B6, C57BL/6J; DBA, DBA/2J; NZW, NZW/LacJ; SJL, SJL/J; F, female; M, male; EC_50_, the concentration required to produce half‐maximal relaxation (−log [*M*]); *p*‐values, two‐way ANOVA main effect *p*‐values for strain, sex, and strain × sex interaction; differences, results of multiple comparison tests. *p*‐values <0.05 are indicated in bold.

### 3.2 Endothelium‐independent relaxation to SNP

**FIGURE 3:**
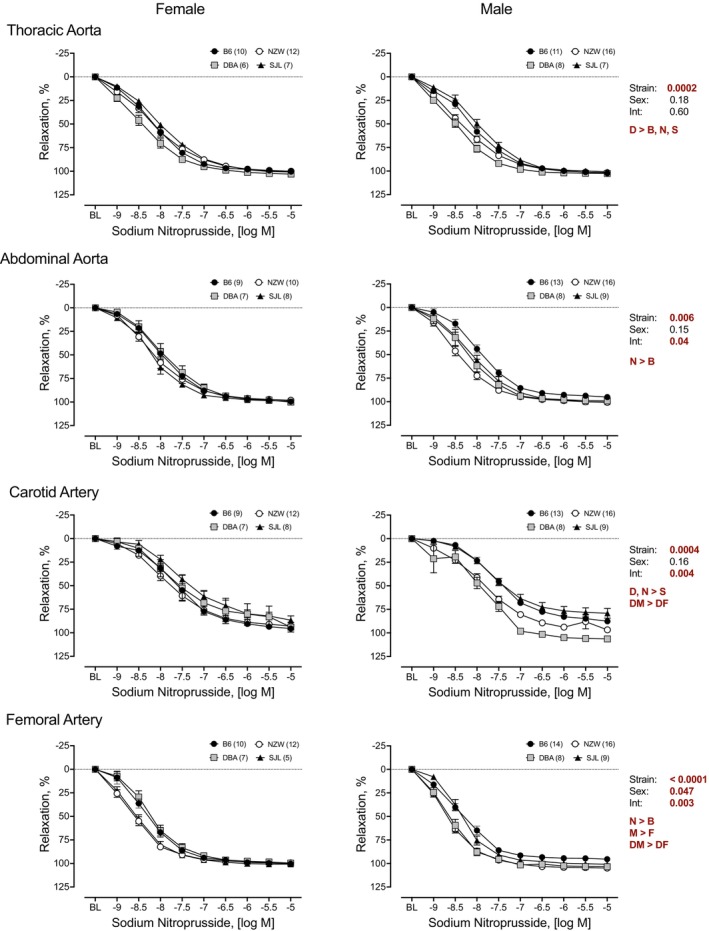
Strain and sex differences in endothelium‐independent relaxation to sodium nitroprusside (SNP) in thoracic and abdominal aorta, and carotid and femoral arteries. Cumulative concentration–response curves to SNP (10^−9^ to 10^−5^ M) were assessed in isolated arteries from female (*left*) and male (*right*) mice from four inbred strains, C57BL/6J (B6), DBA2/J (DBA), NZW/LacJ (NZW), and SJL/J (SJL). Cumulative concentration–response curves are expressed as percent relaxation (%). Main effects for strain, sex, and their interaction from a factorial repeated measures analysis are shown for each artery. The *p*‐value for concentration was significant for each artery (*p* < 0.0001 for all). Strain differences, determined by Tukey post hoc analysis, are indicated by letters B, B6; D, DBA; N, NZW; S, SJL. Sex differences are indicated by M, male; F, female. Males and females are plotted separately for clarity. Numbers in parentheses indicate the number of animals per strain for each artery. Values are expressed as mean ± SEM.

**FIGURE 4:**
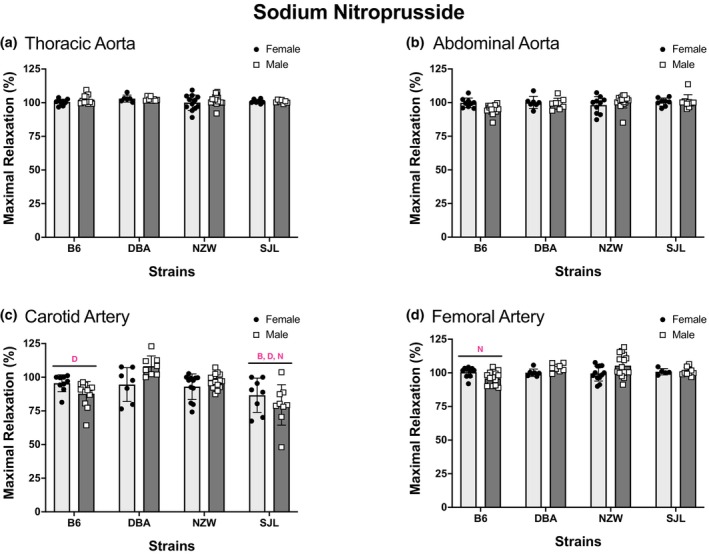
Strain differences in maximal responses to sodium nitroprusside. Values are expressed as mean ± SEM. Two‐way analysis of variance was used to determine the main effects of strain, sex, and interaction for (a) thoracic aorta (*p*_strain_ = 0.3, *p*_sex_ = 0.4, *p*_int_ = 0.7), (b) abdominal aorta (*p*_strain_ = 0.2, *p*_sex_ = 0.5, *p*_int_ = 0.07), (c) carotid artery (*p*_strain_ ≤ 0.0001, *p*_sex_ = 0.8, *p*_int_ = 0.005), and (d) femoral artery (*p*_strain_ = 0.03, *p*_sex_ = 0.3, *p*_int_ = 0.005). Strain differences, determined by Tukey post hoc analysis, are indicated by letters B, B6; D, DBA; N, NZW. The number of animals per strain, sex, and artery is listed in Figure 3.

Endothelium‐independent vasorelaxation was assessed by concentration–response curves to SNP (Figure 3). All arteries from each strain relaxed in response to SNP, but a significant main effect of strain was identified for each artery. Strain differences varied by artery. For TA, AA, and FA, responses in B6 were significantly lower than other strains. In CA, SJL had the lowest response. A significant main effect of sex was observed in FA, with responses in males greater than those in females. Significant strain by sex interactions was identified in the CA and FA (Figure 3). Pairwise comparisons indicated that responses from male DBA mice were greater than those from female DBA mice in both arteries although responses to SNP exceeded 94% in all groups (Figure 4). Two‐way ANOVA identified significant effects of strain in CA and FA for maximal responses to SNP (Figure 4). In CA, maximal responses were smallest in arteries from SJL mice. Average relaxation responses were less than 87% in arteries from SJL mice and greater than 93% in arteries from most other strains. The limited strain differences are reflected in the strain effect sizes (*η* ^2^) for maximal responses to SNP. Effect size was highest in the CA (*η* ^2^ = 0.25) and <0.1 in all other arteries (FA: *η* ^2^ = 0.09, AA: *η* ^2^ = 0.06, TA: *η* ^2^ = 0.05). Sensitivity as determined by SNP EC_50_ (−log_10_ [*M*]) also varied by strain in each artery (Table 2). A significant effect of strain was observed for all arteries. In each artery, the sensitivity to SNP was greater in NZW than at least one other strain. No significant sex differences for SNP EC_50_ were observed for any artery tested (Table 2).

**TABLE 2:** Half‐maximal relaxation concentrations (EC_50_) for sodium nitroprusside (SNP).

Vessel/strain	Female	Male	*p*‐Value	Differences
Thoracic aorta				
B6 (10/11)	8.25 ± 0.08	8.29 ± 0.12	Strain: **0.0009**	DBA, NZW > SJL
DBA (6/8)	8.65 ± 0.16	8.67 ± 0.10	Sex: 0.44	DBA > B6
NZW (12/15)	8.36 ± 0.16	8.59 ± 0.13	Int.: 0.76	
SJL (7/9)	8.07 ± 0.06	8.06 ± 0.11		
Abdominal aorta				
B6 (9/12)	8.03 ± 0.11	7.95 ± 0.07	Strain: **0.01**	NZW > B6
DBA (7/8)	7.95 ± 0.15	8.34 ± 0.17	Sex: 0.10	
NZW (10/14)	8.20 ± 0.12	8.45 ± 0.08	Int.: 0.11	
SJL (8/9)	8.19 ± 0.07	8.15 ± 0.10		
Carotid artery				
B6 (9/13)	7.74 ± 0.15	7.66 ± 0.10	Strain: **0.003**	DBA, NZW > SJL
DBA (6/7)	7.88 ± 0.09	7.95 ± 0.15	Sex: 0.49	
NZW (12/15)	7.91 ± 0.11	7.94 ± 0.08	Int.: 0.65	
SJL (8/9)	7.45 ± 0.12	7.66 ± 0.03		
Femoral artery				
B6 (10/14)	8.44 ± 0.11	8.44 ± 0.10	Strain: **0.01**	NZW > B6
DBA (7/8)	8.28 ± 0.13	8.71 ± 0.12	Sex: 0.91	
NZW (11/14)	8.77 ± 0.13	8.74 ± 0.08	Int.: **0.03**	
SJL (5/9)	8.72 ± 0.10	8.36 ± 0.07		

*Note*: Data are mean ± SEM; numbers in parentheses equal the number of females and males; B6, C57BL/6J; DBA, DBA/2J; NZW, NZW/LacJ; SJL, SJL/J; EC_50_, the concentration required to produce half‐maximal relaxation (−log [*M*]); *p*‐values, two‐way ANOVA main effect *p*‐values for strain, sex, and strain × sex interaction; differences, results of multiple comparison tests. *p*‐values <0.05 are indicated in bold.

### 3.3 l‐NAME effect on relaxation

**FIGURE 5:**
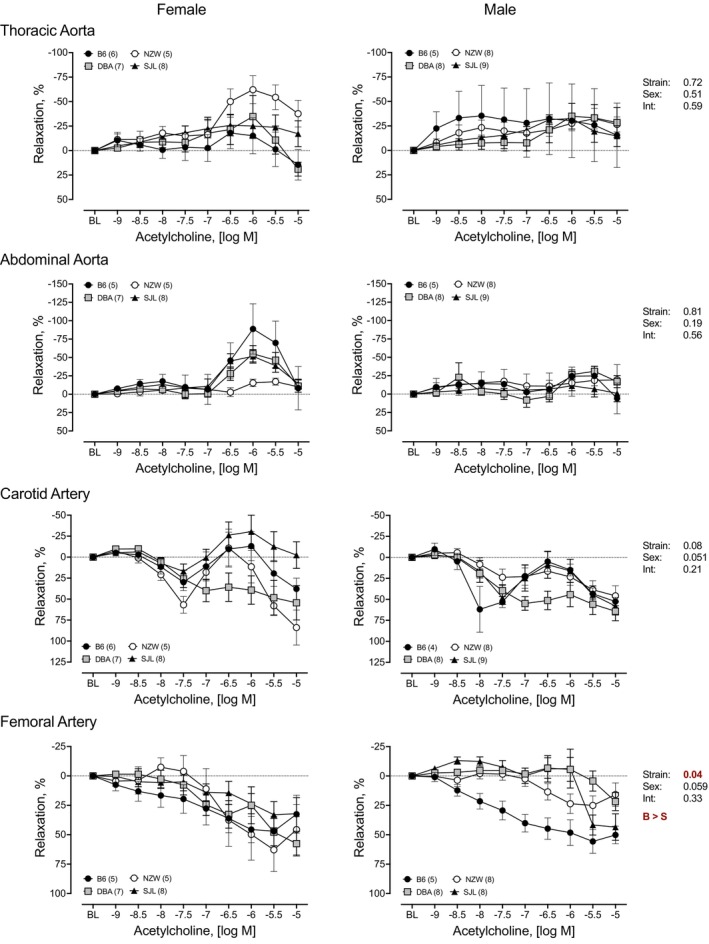
Effect of l‐NAME on relaxation responses to acetylcholine (ACh) in thoracic and abdominal aorta, and carotid and femoral arteries. Cumulative concentration–response curves to ACh (10^−9^ to 10^−5^ M) after incubation with l‐NAME (10^−4^ M, 30 min) were assessed in isolated arteries from female (*left*) and male (*right*) mice from four inbred strains, C57BL/6J (B6), DBA2/J (DBA), NZW/LacJ (NZW), and SJL/J (SJL). Cumulative concentration–response curves are expressed as percent relaxation (%). Negative numbers indicate contraction. Main effects for strain, sex, and their interaction from a factorial repeated measures analysis are shown for each artery. The *p*‐value for concentration was significant for each artery (*p* < 0.0001 for all). Strain differences, determined by Tukey post hoc analysis, are indicated by letters B, B6; S, SJL. Males and females are plotted separately for clarity. Numbers in parentheses indicate the number of animals per strain for each artery. Values are expressed as mean ± SEM. l‐NAME, *N* omega‐nitro‐l‐arginine methyl ester hydrochloride.

**FIGURE 6:**
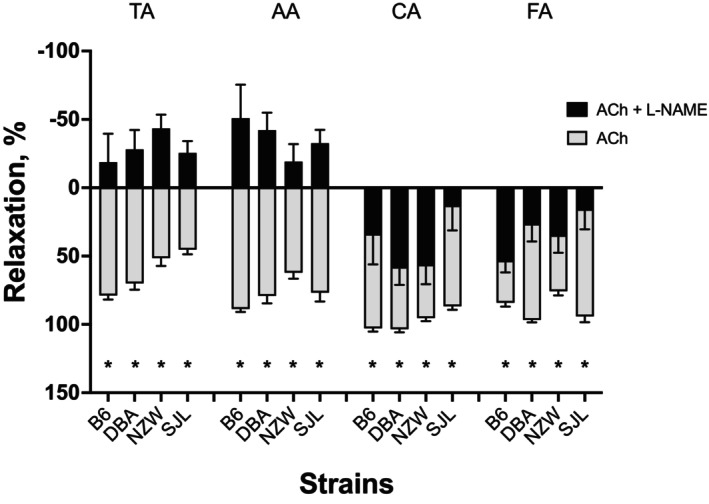
Inhibiting endothelial nitric oxide synthase (eNOS) alters maximal relaxation responses to acetylcholine (ACh) in an artery‐dependent manner. Responses to ACh were obtained in the absence (ACh, gray bars) or presence of l‐NAME (ACh + l‐NAME, black bars) from the thoracic (TA) and abdominal (AA) aorta, and carotid (CA) and femoral (FA) arteries. Arteries from C57BL/6J (B6), DBA2/J (DBA), NZW/LacJ (NZW), and SJL/J (SJL) were incubated with l‐NAME (10^−4^ M, 30 min) prior to generating concentration–responses curves (Figure 5). Male and female data for each condition per strain are combined for each artery. Values are expressed as mean ± SEM. *p < 0.05 ACh + l‐NAME is significantly different from ACh. l‐NAME, *N* omega‐nitro‐l‐arginine methyl ester hydrochloride.

Concentration–response curves to ACh in the presence of l‐NAME are shown in Figure 5. l‐NAME abolished relaxation responses in the TA and AA, and reduced relaxation responses in the CA and FA (Figures 5 and 6). In all strains and arteries, the maximal responses to ACh with l‐NAME were significantly different from those without l‐NAME (Figure 6). After incubation with l‐NAME, ACh elicited contractions in the TA and AA (Figures 5 and 6). Contractions were >15% in all strains in TA and AA. There were no significant strain or sex differences in the responses to ACh in the presence of l‐NAME in the TA. In the AA, a two‐way ANOVA revealed maximal responses (e.g., contraction) to ACh in the presence of l‐NAME were significantly greater in aorta of female mice (female: −54.8 ± 9.9%, male: −18.1 ± 9.0%, *p* = 0.009). In contrast to the TA and AA, the CA and FA were still able to relax in the presence of l‐NAME (Figures 5 and 6). However, maximal relaxation was reduced. These data indicate that the TA and AA rely more on NO‐mediated relaxation than CA and FA in all strains of mice studied. The greatest effect of l‐NAME on CA relaxation was observed in SJL, relaxing <20% of the maximal response to ACh without l‐NAME (Figure 6). CA from NZW and DBA were able to relax to roughly 60% of the maximal response after l‐NAME, about 40% less than when NOS was not inhibited. In the FA, ACh elicited small relaxation responses in the presence of l‐NAME that were 20%–60% of the responses to ACh without l‐NAME (Figures 5 and 6). There was a significant effect of strain in the FA; concentration‐dependent responses in B6 were significantly greater than those in SJL. Two‐way ANOVA also revealed a significant effect of sex for maximal responses to ACh with l‐NAME present. Relaxation responses in FA were significantly greater in females (49.8 ± 8.5%) than in males (20.8 ± 7.9%, *p* = 0.02).

### 3.4 Vasoconstrictor responses to PE

**FIGURE 7:**
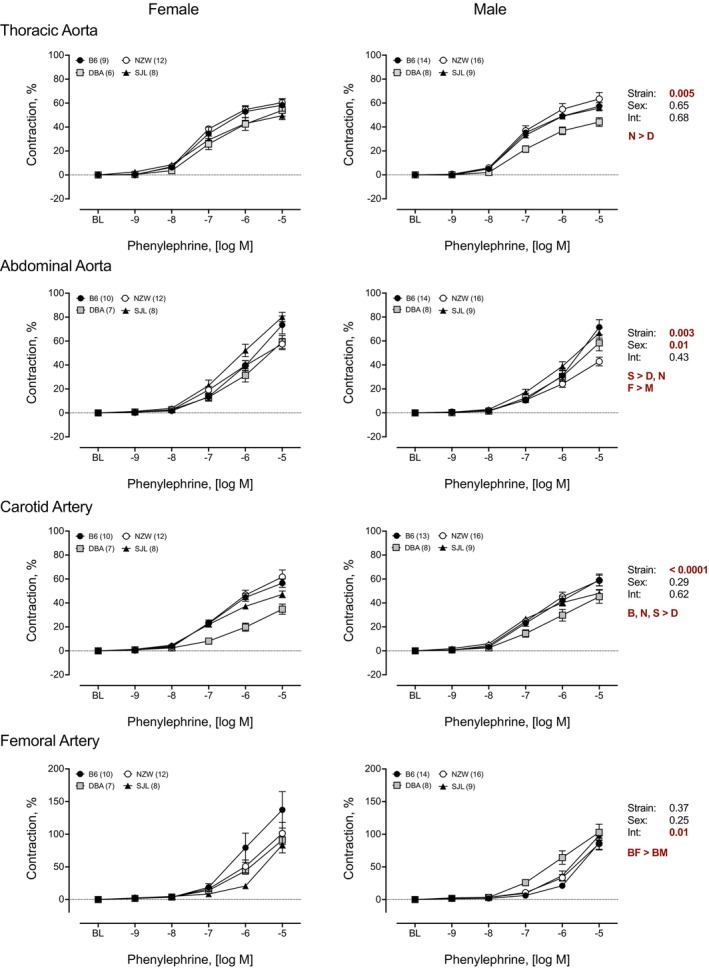
Strain and sex differences in contractile responses to phenylephrine (PE) in thoracic and abdominal aorta, and carotid and femoral arteries. Cumulative concentration–response curves to PE (10^−9^ to 10^−5^ M) were assessed in isolated arteries from female (*left*) and male (*right*) mice from four inbred strains, C57BL/6J (B6), DBA2/J (DBA), NZW/LacJ (NZW), and SJL/J (SJL). Cumulative concentration–response curves are expressed as percent relaxation (%). Main effects for strain, sex, and their interaction from a factorial repeated measures analysis are shown for each artery. The *p*‐value for concentration was significant for each artery (*p* < 0.0001 for all). Strain differences, determined by Tukey post hoc analysis, are indicated by letters B, B6; D, DBA; N, NZW; S, SJL. Sex differences are indicated by M, male; F, female. Males and females are plotted separately for clarity. Numbers in parentheses indicate the number of animals per strain for each artery. Values are expressed as mean ± SEM.

**FIGURE 8:**
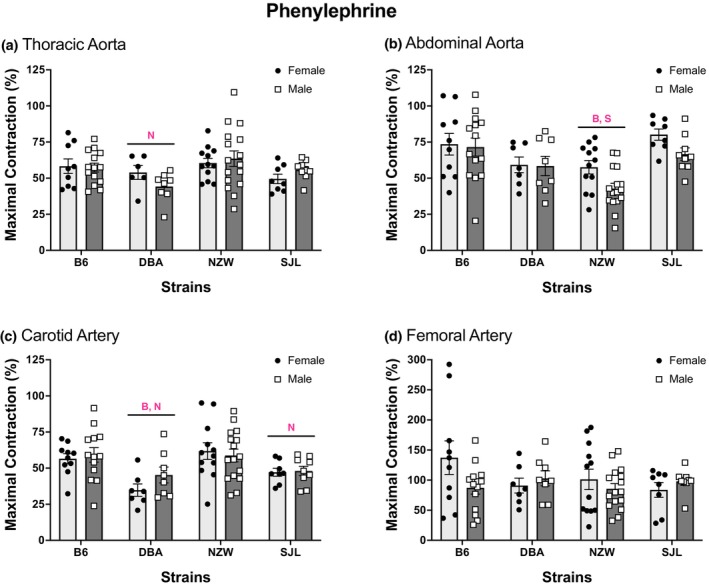
Maximal responses to phenylephrine differ by strain. Values are expressed as mean ± SEM. Two‐way analysis of variance was used to determine the main effects of strain, sex, and interaction for (a) thoracic aorta (*p*_strain_ = 0.03, *p*_sex_ = 0.9, *p*_int_ = 0.04), (b) abdominal aorta (*p*_strain_ ≤ 0.0001, *p*_sex_ = 0.058, *p*_int_ = 0.4), (c) carotid artery (*p*_strain_ = 0.0003, *p*_sex_ = 0.4, *p*_int_ = 0.6), and (d) femoral artery (*p*_strain_ = 0.4, *p*_sex_ = 0.4, *p*_int_ = 0.1). Strain differences, determined by Tukey post hoc analysis, are indicated by letters B, B6; N, NZW; S, SJL. The number of animals per strain, sex, and artery is listed in Figure 7.

Contractile responses to the α_1_ adrenergic agonist PE varied across the strains tested. Concentration–response curves are shown in Figure 7. Significant strain effects were identified for TA, AA, and CA. Contractile responses in arteries from DBA mice were lowest in TA and CA, whereas NZW had the smallest contractions in AA. This pattern was also observed for maximal responses to PE (Figure 8). Two‐way ANOVA detected significant strain differences in maximal responses in TA, AA, and CA (Figure 8). In general, contractile responses were low in arteries from DBA and high in arteries from B6 mice. In TA and CA responses in DBA were approximately 20% less than the highest responding strain. The effect of strain was larger for AA (*η* ^2^ = 0.27) and CA (*η* ^2^ = 0.21) than TA (*η* ^2^ = 0.17) or FA (*η* ^2^ = 0.10). There were no significant sex differences identified by two‐way ANOVA. Significant differences in AUC were found in TA, AA, and CA (Table 3), with large effects for TA (*η* ^2^ = 0.21) and CA (*η* ^2^ = 0.39) and moderate effects of strain for AA (*η* ^2^ = 0.10) and FA (*η* ^2^ = 0.08). The pattern of strain differences for AUC was similar to other measures of PE‐induced contraction. AUC was significantly lower in TA, AA, and CA from DBA compared to other strains, whereas AUC from NZW was significantly less than AUC from SJL in AA (Table 3). Significant sex differences for AUC were observed in AA. Females had greater responses than males. Sex differences in AUC were not identified in the other arteries tested.

**TABLE 3:** Area under the curve (AUC) for phenylephrine.

Vessel/strain	Female	Male	*p*‐Value	Differences
Thoracic aorta				
B6 (9/14)	123.5 ± 14.3	118.1 ± 6.2	Strain: **0.003**	B6, NZW > DBA
DBA (6/8)	99.4 ± 12.5	82.5 ± 7.8	Sex: 0.39	
NZW (12/15)	130.5 ± 5.0	122.1 ± 9.6	Int.: 0.65	
SJL (8/9)	107.3 ± 8.4	115.5 ± 4.2		
Abdominal aorta				
B6 (10/14)	92.0 ± 9.5	79.8 ± 8.8	Strain: **0.008**	SJL > DBA, NZW
DBA (7/8)	77.4 ± 11.1	74.0 ± 14.4	Sex: **0.009**	F > M
NZW (12/16)	90.3 ± 8.9	58.9 ± 6.2	Int.: 0.46	
SJL (8/9)	120.0 ± 12.1	92.8 ± 8.5		
Carotid artery				
B6 (10/13)	100.4 ± 8.5	98.4 ± 9.0	Strain: **<0.0001**	All > DBA
DBA (7/8)	48.5 ± 6.9	69.9 ± 11.4	Sex: 0.27	
NZW (12/16)	104.4 ± 8.4	104.0 ± 8.6	Int.: 0.59	
SJL (8/9)	88.6 ± 6.0	98.5 ± 8.0		
Femoral artery				
B6 (8/14)	117.6 ± 25.2	73.7 ± 7.0	Strain: 0.31	
DBA (7/7)	108.6 ± 12.8	130.2 ± 16.1	Sex: 0.49	
NZW (12/16)	123.6 ± 21.8	91.7 ± 10.0	Int.: 0.07	
SJL (8/9)	77.8 ± 13.6	100.8 ± 12.5		

*Note*: Data are mean ± SEM; numbers in parentheses equal the number of females and males; B6, C57BL/6J; DBA, DBA/2 J; NZW, NZW/LacJ; SJL, SJL/J; F, female; M, male; AUC, area under the curve (%*x* – log [*M*]), *p*‐values, two‐way ANOVA main effect *p*‐values for strain, sex, and strain × sex interaction; differences, results of multiple comparison tests. *p*‐values <0.05 are indicated in bold.

## 4 DISCUSSION

The aims of this study were to (1) assess the influence of genetic background on vasomotor function in arteries from different locations in the arterial tree; (2) identify sex differences in vasomotor function; and (3) determine the importance of NO in vasorelaxation response in male and female mice with different genetic backgrounds. Male and female mice were studied to determine sex differences within strain. In addition, we determined whether the reliance of these arteries on NO‐mediated vasorelaxation varied by artery and strain. We hypothesized that NO‐mediated vasorelaxation in the AA, CA, and FA would be impaired in strains with previously documented impairment in the TA. Furthermore, we hypothesized that NO‐mediated vasorelaxation would be impaired in arteries from male mice when compared to arteries from female mice. The primary findings of this study are as follows: (1) Strain differences existed in relaxation responses to ACh for all arteries. B6 and DBA had greater relaxation responses than NZW and SJL. Sensitivity differences (EC_50_) were found in AA, CA, and FA with NZW displaying decreased responsiveness in relation to the other strains. In contrast, maximal responses to the endothelium‐independent vasodilator SNP only differed by strain in the CA and FA. (2) Maximal contractile responses to PE varied across strains in all arteries except the FA. The strain distribution pattern was similar in TA and CA and varied in AA and FA. (3) In the presence of a NOS inhibitor, l‐NAME, relaxation responses to ACh were impaired in all arteries and resulted in contraction in the TA and AA. (4) There were minimal sex differences in the contractile and relaxation responses across vessels from the four strains included in this study. Overall, these data provide evidence for the influence of genetic background on vasomotor function in multiple arteries. Conversely, biological sex did not significantly influence overall vasomotor function in healthy adult inbred mice.

### 4.1 Endothelium‐dependent vasorelaxation

Our laboratory previously reported differences in endothelium‐dependent relaxation to ACh in TA from 27 different strains of male mice (Kim et al., 2016). In the current study, four strains of male and female mice were used to assess endothelial function in multiple arteries based on their impaired responses in the TA. Also, all of the included strains have been used to investigate some aspect of CVD (e.g., vascular remodeling or atherosclerosis) (Korshunov & Berk, 2004; Paigen et al., 1985, 1990; Rau et al., 2015; Wang et al., 2019). Overall, genetic background or mouse strain significantly affects relaxation responses to ACh in all vessels tested. Approximately, 17%–29% of the variation in relaxation responses to ACh can be attributed to mouse strain based on calculated effect size (*η* ^2^). Strain differences in response to ACh were observed in TA which concurs with our previous findings and others, (Chen et al., 2007; Kim et al., 2016; Ryan et al., 2002; Steppan et al., 2020).

To extend these findings to other vessels, vasorelaxation responses were also assessed in the AA, CA, and FA to determine whether strain differences identified in the TA were present in other arteries. Significant strain differences were observed for every artery. The pattern of strain differences in the AA and CA was like those in the TA suggesting that genetic background has a similar influence on vasorelaxation responses in these arteries. For all arteries studied, concentration‐dependent and/or maximal responses to ACh were significantly lower in NZW compared to the other strains. The sensitivity to ACh was also significantly lower in arteries from NZW mice (Table 1). Furthermore, relaxation responses to ACh (ACh Max, %) were markedly lower in NZW TA (54%) and AA (65%) in comparison with their CA (95%) and FA (76%). When comparing TA and AA to CA or FA, the difference between maximal responses was greater than 40%. This large range of responsiveness to ACh was similar in SJL, but not observed in other strains. In combination, these results suggest that endothelium‐dependent relaxation is impaired in NZW mice relative to other strains and is not localized to the TA. Relaxation responses in SJL also were low for TA and CA. The impaired responses in the TA are similar to those reported previously by us (Chen et al., 2007; Kim et al., 2016) and not different from those in NZW mice. In CA, relaxation responses were significantly lower in arteries from SJL compared with all other strains (Figures 1 and 2). The strain differences in endothelium‐dependent relaxation in the current study contrasts with Ryan et al. (2002) who previously reported responses to ACh were similar in CA from three inbred strains of mice, despite significant differences in endothelial function in aorta. It is important to note however the only strain common to both studies was the B6, which was not found to have impaired function in either study. In most strains, higher doses of ACh induced transient contractions in the CA suggesting activation of receptors on the vascular smooth muscle or ACh‐induced release of contracting factors from the endothelium. Previous research demonstrated that ACh initiates competing pathways in AA, CA, and FA (Zhou et al., 2005). Specifically, endothelium‐dependent relaxation is mediated primarily by NO and endothelium‐dependent contraction has been proposed to be mediated by thromboxane A2 (Kobayashi et al., 2004). Traupe et al. (2002) also reported endothelium‐dependent contractions to higher concentrations of ACh in mouse CA mediated by the prostaglandin H2/thromboxane A2 pathway. Like Traupe et al., we did not observe ACh‐induced contraction in other arteries suggesting that the prostaglandin pathway is more prominent in the CA than in the other arteries tested and varied somewhat by mouse strain. It is also worth noting that in arterial rings denuded of their endothelium that ACh can cause contraction of the vascular smooth muscle via the activation of muscarinic type 2 (M2) receptors. When the endothelium is present, this contraction induced by administration of ACh is overshadowed by endothelium‐derived relaxing factors such as NO, prostacyclin, and hyperpolarizing factors (Jaiswal et al., 1991; Zygmunt et al., 1994). It is possible the biphasic nature of the CA is due to a combination of increased endothelium‐derived contracting factors and greater abundance of M2 receptors on the vascular smooth muscle.

### 4.2 Endothelium‐independent vasorelaxation

Similar to responses to ACh, there were strain differences for each artery in response to the endothelium‐independent agent, SNP. For concentration‐response curves, the strain differences were not consistent across arteries; however, responses in DBA and NZW were generally greater than the other strains. Strain differences were also observed for maximal responses in CA and FA with responses being greater in DBA and NZW. The near 100% relaxation responses to the endothelium‐independent agent SNP in all arteries from NZW mice suggest that the impaired responses to ACh in NZW arteries are not related to impaired vascular smooth muscle function. Therefore, future studies of ACh‐mediated relaxation in NZW mice should focus on ACh‐signaling pathways and factors affecting NO bioavailability. In contrast, relaxation responses to SNP were significantly reduced in CA from SJL mice. This reduction in the CA implies that the impaired responses to ACh in CA from SJL might be due, in part, to altered vascular smooth muscle and not just endothelial dysfunction. Impaired endothelial and vascular smooth muscle function in CA from SJL is consistent with the significant inward remodeling that occurs in this strain in response to low flow following complete or partial ligation of the CA (Harmon et al., 2000; Korshunov & Berk, 2004).

### 4.3 NO inhibition

In the present study, the NOS inhibitor l‐NAME was used to determine the reliance of arteries on NO‐mediated relaxation. Incubation with l‐NAME had a significant inhibitory effect on all the arteries tested regardless of strain or sex of mouse. This effect was greatest in the large, elastic or conducting arteries (TA and AA), which contracted in response to ACh after NOS inhibition. The contractions in the TA and AA suggest that NO is the primary mechanism for ACh‐induced relaxation in those arteries. In studies utilizing either eNOS^−/−^ mice or wild type mice under the influence of l‐NAME, endothelium‐dependent contractions have been reported in AA, CA, and FA (Crauwels et al., 2000; Williams et al., 2014; Zhang et al., 2001; Zhou et al., 2005). These contractions were blunted when a cyclooxygenase (COX) inhibitor indomethacin, thromboxane A2, and/or prostaglandin H_2_ receptor antagonist were introduced (Tang et al., 2005; Williams et al., 2014). Collectively, these findings suggest that ACh activates competing endothelium‐derived relaxing (NO) and contracting factors (prostaglandin H_2_/thromboxane A_2_). In contrast to aortic segments, relaxation responses to ACh were partially inhibited in the CA and FA (Figures 5 and 6). Partial inhibition of ACh relaxations in FA and complete inhibition in TA from rats has been reported previously (Nagao et al., 1992). In contrast, Crauwels et al. (2000) reported complete inhibition of relaxation to ACh in CA and partial inhibition in FA from female B6 mice. The residual relaxation in the FA was attributed to a factor other than NO or prostacyclin (Crauwels et al., 2000). The administration of ACh can cause vasodilation independent of NO. Zygmunt et al. (1994) demonstrated an ACh‐induced, endothelium‐dependent vascular relaxation despite utilizing a NOS and COX inhibitor. This relaxation was abolished in endothelium‐denuded vessels, and in the presence of 30 mM K^+^ solution. In that study, interference with NOS, COX, hyperpolarization, and the endothelium shows that ACh can cause NO‐independent vascular relaxation that is likely the result of endothelium‐derived hyperpolarizing factor. The disparity in response to ACh reported here and by others (Crauwels et al., 2000; Nagao et al., 1992; Zygmunt et al., 1994) across arteries of varying size and location suggests potential differences in reliance on competing endothelium‐derived relaxing and contracting factors. Despite differences in the effect of l‐NAME on ACh responses across arteries, the only significant strain differences in maximal responses were observed on the FA. The lack of strain differences in the other arteries in responses to ACh in the presence of l‐NAME supports the concept that a difference in NO bioavailability is the likely mechanism underlying strain differences in response to ACh alone.

### 4.4 Vasocontraction

We also identified significant strain‐dependent differences in contractile responses to PE in TA, AA, and CA (Figures 7 and 8). Strain effects accounted for 10%–25% of the variation in maximal responses to PE in those arteries. The strain distribution pattern was similar in the TA and CA with greater responses in NZW and B6 and smaller responses in DBA. This strain distribution matches our previous study in TA from male mice of those strains (Kim et al., 2016). In contrast, results were mixed in other strain comparisons of contractile responses. Some found no strain differences (Steppan et al., 2020) or strain differences with responses in B6 being higher (Steppan et al., 2020) or lower (Ryan et al., 2002; Steppan et al., 2020) than the other strains tested depending on the artery and agonist utilized and strains included. Regardless of strain, contractions to PE were consistently the greatest in the FA. A similar pattern of responses between arteries was reported in B6 mice (Kleinbongard et al., 2013). In seven different arterial segments from B6 mice, absolute changes in tension in response to KCl and NE were greater in AA and FA relative to TA and CA. A similar pattern was observed for responses to 10 μM PE and 50 mM K^+^ in TA, CA, and FA from B6 mice (Leloup et al., 2015). Several factors could contribute to differences in contractile responses between strains for a specific vessel and between vessels, including structural differences between muscular and elastic arteries and variation in α_1_ adrenergic receptor function and number. There is evidence supporting regional variation in PE‐induced contractions based on receptor subtypes both between vessels and within regions of larger vessels (Yamamoto & Koike, 2001). However, the information regarding strain differences in adrenergic receptor number and function is limited.

### 4.5 Sex differences

We hypothesized that vasomotor function would differ between arteries from male and female mice. Contrary to our hypothesis, there were few sex differences in relaxation responses to ACh or contractile responses to PE. Concentration‐response curves to ACh differed by sex in AA and CA, with females having greater responses in AA and smaller responses in CA. The only strain‐specific sex differences were observed in relaxation responses to ACh in the TA from NZW mice and FA of SJL mice. The impaired responses in male NZW concur with our previous finding (Kim et al., 2016). Responses in aorta from female NZW mice were greater than those in males and comparable to females from the other strains, suggesting that the mechanism for the impaired relaxation response is male‐specific. Typically, male NZW mice are used to generate a mouse model of lupus (NZBW F1). Female mice from that F1 strain can develop impaired endothelium‐dependent relaxation later in life (e.g., >20 weeks old) compared to female NZW mice (Ryan & McLemore, 2007), suggesting that the phenotype might be somewhat heritable. In addition, it is unclear whether sex differences in inbred NZW mice persist or become larger with aging as responses in male NZW or NZBW F1 mice are generally not reported. Sex differences were also observed for concentration‐dependent relaxation responses to SNP in CA and FA from DBA mice. Responses in vessels from female mice were reduced compared to those from male mice. Although the mechanism for the reduced responses is not clear, maximal relaxation responses were ≥94% in all groups suggesting that vascular smooth muscle function is not impaired in any of the groups.

The limited number of sex differences across the other strains and arteries might be related to the age of the mice included in this study. Sex differences might not be as readily apparent in the young adult mice included in this study. Although sex differences in endothelial function have been reported in younger mice (Faulkner et al., 2021; Ogola et al., 2022; Padilla et al., 2019), those findings are not consistent (Cole et al., 2022; Takenouchi et al., 2009; Wu et al., 2022). Greater sex differences might have been observed if mature adult mice were included in the current study. However, studies in B6 mice ranging in age from 4 to 9 and 23 to 32 months old reported no sex differences in vasomotor function in mesenteric arteries (Cole et al., 2022). In addition, no differences were observed in thoracic and AAs from 6‐week‐old mice (Wu et al., 2022). Therefore, sex differences in vasomotor function in healthy inbred mice might be strain and vessel specific.

### 4.6 Limitations and conclusions

Our study has a few limitations. Although estrogen can influence vascular function, we did not measure circulating estrogen nor monitor the estrous cycle in our female mice. Recent evidence suggests that the estrous cycle does not influence vasomotor function in blood vessels isolated from female B6 mice (Kehmeier et al., 2022). However, future studies should be cognizant of the influence circulating estrogen and the estrous cycle might have on vascular function. We identified significant strain differences in vasomotor function, suggesting that genetic differences contribute to variation in vasomotor control. However, the genes/genetic factors underlying these differences are unclear. The amount of tissue available after processing the vessels for functional studies was not adequate to explore potential mechanisms for these differences. Based on our previous study (Chen et al., 2007), differences in NO signaling and/or redox regulatory pathways likely contribute to some of these strain differences. Finally, all the vessels included in this study can be considered conduit vessels. It is unclear if sex and strain differences would have been more apparent had small arteries or arterioles been included in the current study.

In summary, the results of this study demonstrate that strain differences exist in vasomotor function in large arteries. Strain differences were identified for endothelium‐dependent relaxation to ACh in all arteries and for maximal endothelium‐independent responses to SNP in CA and FA. Contractile responses to PE also showed significant strain differences, although the differences were not as uniform across arteries. B6 mice consistently displayed relatively high maximal contractile responses to PE and relaxation responses to ACh, with average maximal relaxation responses greater than 85% across all arteries. In contrast, relaxation responses in arteries from NZW and SJL were significantly reduced relative to B6 mice. These strain differences add additional evidence that genetic background influences vasomotor function throughout the large arterial vasculature. Our findings also suggest heterogeneity in vascular genotype–phenotype relationship throughout the large, arterial network. Relative to other arteries, the CA showed a unique strain‐dependent pattern of responses for endothelium‐dependent and independent relaxation. In addition, NOS inhibition was least effective for reducing responses to ACh in the CA and FA. This suggests alternative pathways for relaxation are more prominent in those arteries. The strain and artery differences observed in the present study reinforce the concept that arteries and mouse strains should be selected carefully for studies of vascular function and vascular disease.

## AUTHOR CONTRIBUTIONS

All authors listed made a substantial, direct, and intellectual contribution to the work, and approved it for publication. Michael P. Massett supervised and coordinated the study. Dylan Holly and Hyoseon Kim performed all experiments. Michael P. Massett, Dylan Holly, and Hyoseon Kim analyzed the data. Dylan Holly, Michael P. Massett, Hyoseon Kim, and Christopher R. Woodman wrote and/or revised the manuscript.

## FUNDING INFORMATION

This work was supported by a Texas A&M Triads for Transformation grant (Michael P. Massett, Christopher R. Woodman), College of Education and Human Development Strategic Research Award (Dylan Holly), Huffines Institute Student Research Grant (Dylan Holly), and Texas A&M Merit Fellowship (Dylan Holly).

## CONFLICT OF INTEREST STATEMENT

The authors declare that the research was conducted in the absence of any commercial or financial relationships that could be construed as a potential conflict of interest.

## ETHICS STATEMENT

Prior to initiating this study, approval was received from the Texas A&M University Institutional Animal Care and Use Committee. All procedures were performed under the Public Health Service’s Policy on Humane Care and Use of Laboratory Animals guidelines.

## Data Availability

The original contributions presented in the study are included in the article; further inquiries can be directed to the corresponding author/s.
